# Integrative analysis of DNA copy number, DNA methylation and gene expression in multiple myeloma reveals alterations related to relapse

**DOI:** 10.18632/oncotarget.13025

**Published:** 2016-11-02

**Authors:** Patryk Krzeminski, Luis A. Corchete, Juan L. García, Lucía López-Corral, Encarna Fermiñán, Eva M. García, Ana A. Martín, Jesús M. Hernández-Rivas, Ramón García-Sanz, Jesús F. San Miguel, Norma C. Gutiérrez

**Affiliations:** ^1^ Departamento de Hematología, Hospital Universitario, IBSAL, IBMCC (USAL-CSIC), Salamanca, Spain; ^2^ Centro de Investigación del Cáncer-IBMCC (USAL-CSIC), Salamanca, Spain; ^3^ Unidad de Genómica y Proteómica, Centro de Investigación del Cáncer-IBMCC (USAL-CSIC), Salamanca, Spain; ^4^ Clínica Universidad de Navarra, Centro de Investigaciones Médicas Aplicadas (CIMA), Pamplona, Spain

**Keywords:** multiple myeloma, DNA methylation, microarrays, SNP, SORL1

## Abstract

Multiple myeloma (MM) remains incurable despite the introduction of novel agents, and a relapsing course is observed in most patients. Although the development of genomic technologies has greatly improved our understanding of MM pathogenesis, the mechanisms underlying relapse have been less thoroughly investigated. In this study, an integrative analysis of DNA copy number, DNA methylation and gene expression was conducted in matched diagnosis and relapse samples from MM patients. Overall, the acquisition of abnormalities at relapse was much more frequent than the loss of lesions present at diagnosis, and DNA losses were significantly more frequent in relapse than in diagnosis samples. Interestingly, copy number abnormalities involving more than 100 Mb of DNA at relapse significantly affect the gene expression of these samples, provoking a particular deregulation of the IL-8 pathway. On the other hand, no significant modifications of gene expression were observed in those samples with less than 100 Mb affected by chromosomal changes. Although several statistical approaches were used to identify genes whose abnormal expression at relapse was regulated by methylation, only two genes that were significantly deregulated in relapse samples (*SORL1* and *GLT1D1*) showed a negative correlation between methylation and expression. Further analysis revealed that DNA methylation was involved in regulating *SORL1* expression in MM. Finally, relevant changes in gene expression observed in relapse samples, such us downregulation of *CD27* and *P2RY8*, were most likely not preceded by alterations in the corresponding DNA. Taken together, these results suggest that the genomic heterogeneity described at diagnosis remains at relapse.

## INTRODUCTION

The survival of patients suffering from multiple myeloma (MM) has improved considerably in the last ten years [1]. However, the clinical course of almost all MM patients is characterized by a chronic recurrence pattern, with periods of remission followed by relapse until the disease eventually becomes refractory [2]. Although MM initially responds readily to treatment and the responses are commonly durable, the time to progression is increasingly short in subsequent relapses. Therefore, further reductions in MM mortality and morbidity depend largely on our ability to prevent, delay or successfully treat the recurrences.

Many advances in our understanding of the pathogenesis of MM have been the result of major developments in genomic technologies [3–5]. However, not enough attention has been paid to identifying the mechanisms that trigger the relapse or progression of MM. A complex clonal architecture has recently been described at diagnosis and at various stages of myeloma progression. Several studies have revealed distinct patterns of subclonal evolution in MM including both linear and branching evolutionary models [6–8]. The contribution of these intraclonal dynamics to disease progression and relapse seems to be essential and may have profound therapeutic implications.

Another important pathogenic mechanism that could be involved in myeloma relapse is the aberrant DNA methylation. A global DNA hypomethylation pattern with selective hypermethylated genes in comparison to normal plasma cells has been described in myeloma cells, providing a possible explanation for the transition from monoclonal gammopathy of undetermined significance (MGUS) to MM [9]. Genomic imbalances and changes in DNA methylation observed at relapse can probably lead to differences in gene expression levels, thereby provoking myeloma relapse or progression. In addition, global hypomethylation induces genomic instability, which may have a greater impact during the late stages of the disease [10].

The need to better understand the differences between the myeloma cells at the time of relapse and those present at diagnosis prompted us to investigate the copy number status, changes in DNA methylation and gene expression under both circumstances, and to integrate the data generated by the three microarray platforms.

## RESULTS

### Different patterns of genomic imbalances at MM relapse

**Figure 1:**
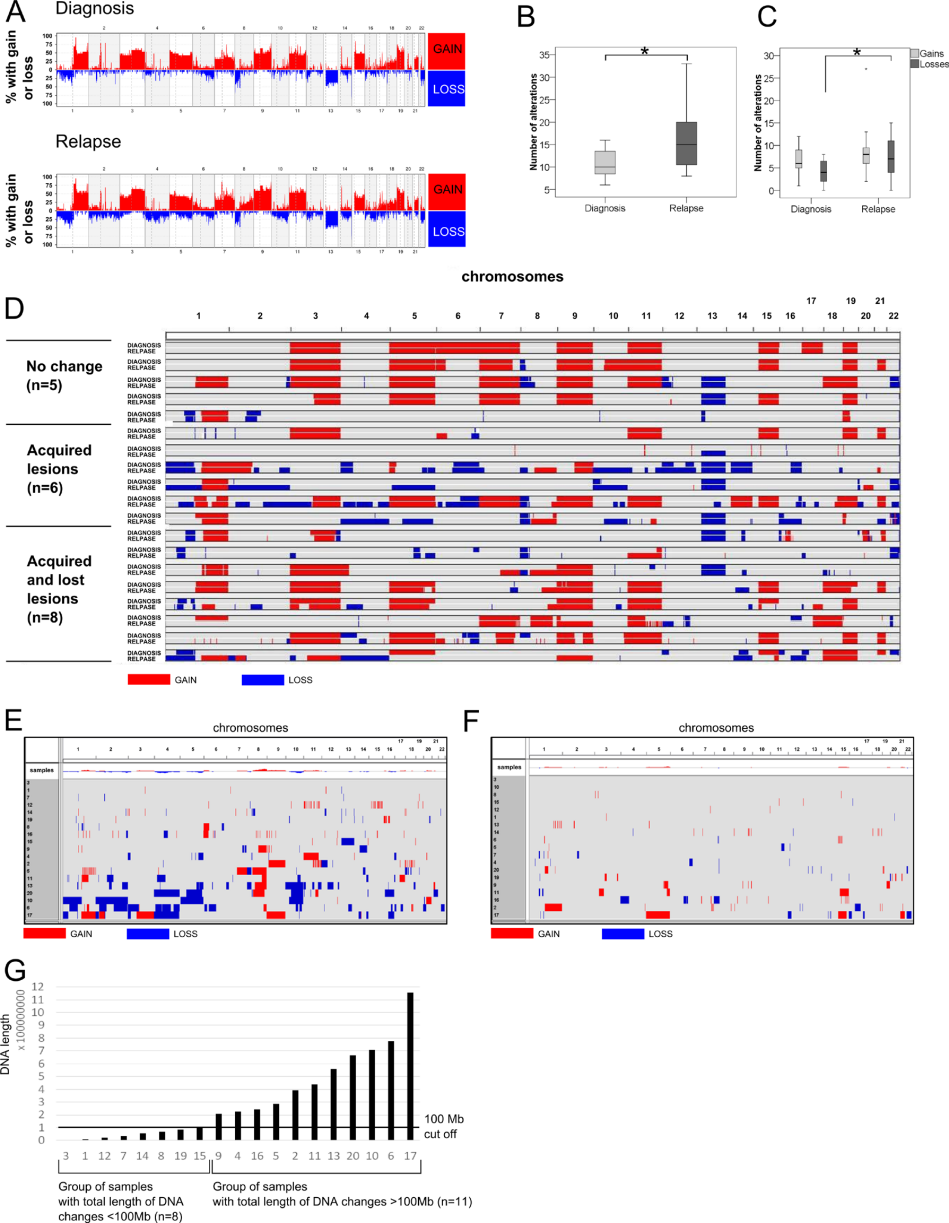
Genomic landscape of MM revealed by SNP microarrays (**A**) Frequency plot of copy number changes (gains and losses) at a chromosomal position in MM samples at diagnosis (*n* = 19) and relapse (*n* = 19). (**B**) Box-plot showing the number of chromosomal changes. *p < 0.01 (Mann–Whitney *U* test). (**C**) Box-plot comparing the number of gains and losses at diagnosis and relapse. *p < 0.01 (Mann–Whitney *U* test). (**D**) Visualization of the size and location of genomic changes comparing diagnosis and relapse. Nineteen paired samples were ordered into three categories: cases with “no change”, “acquired lesions” or “acquired and lost lesions”. Both acquired and lost lesions can refer to gains or losses of chromosomal material. (**E**) Visualization of the size and location of CNAs emerging at relapse and not present at diagnosis. Only new gains and losses are shown. The chromosome number is indicated at the top of the graph. Visualization of the size and location of CNAs present at diagnosis but which had disappeared at relapse. (**G**) Classification of samples according to the total length of changed DNA (gained or lost). The X axis indicates the sample number; the Y axis shows the length of changed DNA (bp). The black line is a 100-Mb cutoff that separates samples into those with small and large DNA changes.

Genomic imbalances were identified in all tested samples obtained at diagnosis and in all the corresponding relapse samples. A summary of all chromosomal changes is presented in Supplementary Table S1 and Figure 1A. Significantly more chromosomal imbalances were observed at relapse with a median of 15 per case (range 8–33) compared with the moment of diagnosis with a median of 10 per case (range 6–16) (*p* = 0.01) (Figure 1B). When gains and losses were considered separately we found that losses were significantly more frequent at relapse (median of 7 per case; range 0–15) than in diagnosis samples (median of 4 per case; range 0–8) (*p* = 0.03) (Figure 1C).

Visual analysis revealed slight differences between diagnosis and relapse in five paired samples. In the remaining cases the diagnosis and relapse samples showed different copy number abnormalities: six pairs only acquired new lesions, while eight pairs acquired new lesions and lost aberrations that were present at diagnosis (Figure 1D). Overall, the acquisition of abnormalities at relapse was much more frequent than the disappearance of lesions present at diagnosis (*p* < 0.002) (Figure 1E and 1F). The most frequently acquired aberrations at relapse and not present at diagnosis were 8q gains and 10q losses (FDR = 0.03 for both abnormalities).

Next, the whole length of DNA affected by copy number abnormalities (CNAs) at relapse in each sample was quantified using the Galaxy subtraction tool. Thus, a set of 11 samples showed a total length of DNA changed by more than 100 Mb at relapse, while CNAs affected less than 100 Mb of DNA in only eight samples (Figure 1G).

### Impact of chromosomal changes at relapse on gene expression of myeloma cells

**Figure 2:**
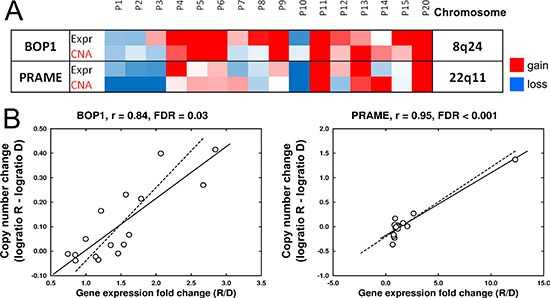
Associations of chromosomal changes and modification of gene expression levels at relapse (**A**) Heatmap showing the significant association between CNA and the expression level of two genes, *BOP1* and *PRAME*. Color scale for expression: blue, Fold Change (FC) R/D < 0.67; red, FC R/D > 1.5; white, FC = 0. Color scale for CNAs: blue, log_2_ ratio R–D < −0.1; red, log_2_ ratio R–D > 0.1; white, log_2_ ratio = 0. R: relapse, D: diagnosis. Data obtained from microarray analysis of 16 paired samples. (**B**) Pearson correlation of CNA and gene expression of *BOP1* and *PRAME*.

To evaluate the influence of specific chromosomal changes at relapse on the modification of the expression levels of the affected genes, a bidirectional correlation analysis between CNAs and gene expression was performed in the 16 paired samples (32 samples in total) with both types of available genomic data. This analysis was restricted to those genes with a ≥ 2-fold change in gene expression in at least three patients. Pearson correlations revealed a positive and significant correlation (*r* > 0.8, FDR < 0.05) for two genes, *PRAME* and *BOP1*, located at 22q11 and 8q24, respectively (Figure 2A and 2B). Gains on 8q24 also contained the *MYC* gene, although the acquisition of this imbalance at relapse was not correlated with *MYC* overexpression. An association between CNAs and gene expression was also sought using a pair-by-pair analysis, but no significant genes were identified by this approach.

**Figure 3:**
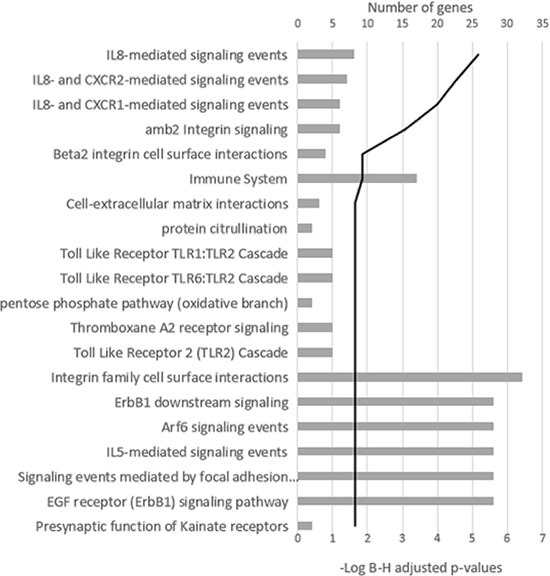
Signaling pathway analysis of the differentially expressed genes in the group of samples with more than 100 Mb of DNA involved in CNAs The 20 most significant pathways are shown. The black line represents the log Benjamini–Hochberg adjusted *p*-value.

When the impact of chromosomal changes on gene expression at relapse was assessed with respect to the length of DNA affected by CNAs, 273 differentially expressed genes between diagnosis and relapse status were detected in the group of samples with more than 100 Mb of DNA involved in CNAs. Signaling pathway analysis classified these genes into 88 pathways (adjusted *p* < 0.05). Those related to cytokines, particularly IL-8, and integrin signaling were the most strongly enriched (Figure 3). On the other hand, no relevant modifications of gene expression were observed in those samples with less than 100 Mb affected by chromosomal changes.

### Changes in DNA methylation between relapse and diagnosis

**Figure 4:**
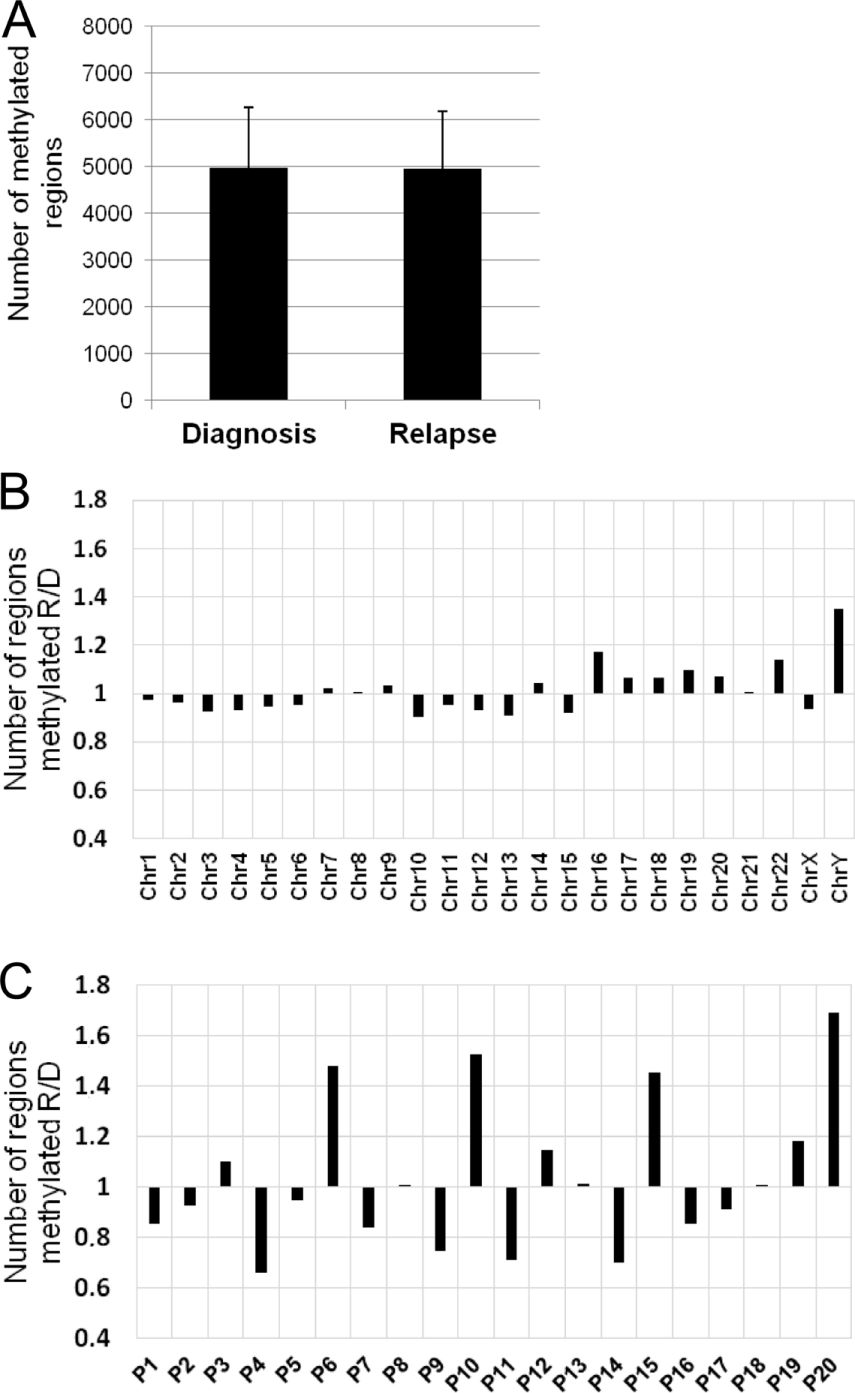
DNA methylation changes between relapse and diagnosis (**A**) Number of methylated regions (mean ± standard deviation) in the 20 paired samples (diagnosis *vs* relapse). (**B**) Ratio of the number of methylated regions between relapse and diagnosis chromosome-by-chromosome. X axis, chromosomes; Y axis, fold change of methylated regions. (**C**) Ratio of the number of methylated regions between relapse and diagnosis for each pair of samples. X axis, sample identification; Y axis, fold change of methylated regions.

As an initial approach to the analysis of changes in DNA methylation between matched diagnosis and relapse samples from 20 MM patients, we measured general methylation status, comparing the number of methylated regions at diagnosis and relapse samples without assigning them to any genes. Overall, no significant differences in the number of methylated regions between MM diagnosis and relapse were found (Figure 4A). In order to clarify this negative result, a pair-by-pair comparison of the number of methylated regions was carried out. This analysis revealed heterogeneous methylation patterns between the paired samples, whereby although most of them showed fewer methylated regions at relapse, seven patients exhibited the opposite pattern (Figure 4B). The chromosome-by-chromosome comparison identified only minute differences (Figure 4C).

Since the analysis revealed no significant differences in the number of methylated regions at diagnosis and relapse, a more detailed DNA methylation analysis was carried out. The analytical strategies are summarized in Supplementary Table S2.

Differentially methylated regions (DMRs) at MM diagnosis and relapse were compared using the Charm R Package. A total of 490 DMRs (*p* < 0.05, average DNA methylation percentage within the DMR > 5%, number of probes more or equal than 4) were identified, whose associated genes were mainly enriched in integrin family members, cell surface interactions and proteoglycan syndecan-mediated signaling events according to Pathway Commons. Interestingly, three members of the cluster of differentiation (*CD7, CD53* and *CD82*), genes involved in the MAPK signaling pathway such as *MAP3K19* and *MAPK8IP3*, the kinase *PI4KB* and the transcriptional factor *POU6F1*, were found among the genes with associated DMRs. The full list of DMRs with associated genes is presented in Supplementary Table S3.

At first sight, analysis of DMR is informative, but further interpretation may be quite complex, since DMR-associated genes are sometimes located thousands of nucleotides away from the methylated sequence, hindering further DNA methylation validation *in vitro*. Thus, the analysis was refined by screening the methylation status of two genomic regions relative to the nearest transcriptional start site (TSS), comparing the states at diagnosis and relapse. The first, 2000 bp upstream TSS considered as a “promoter region” and the second, +/− 250 bp surrounding the TSS called “core promoter”. Additionally, the maximum and average methylation signals were analyzed for each region (Supplementary Figure S1). This approach was dictated by the need to check whether a short sequence in each range could be strongly methylated. The analysis of methylation focused on promoter region showed more than 3000 methylated genes when average methylation was taking into account and 60 when maximum methylation signal was considered (*q* < 0.05). The list of genes identified as having differential DNA methylation levels between diagnosis and relapse is shown in Supplementary Table S4.

### DNA methylation changes at relapse and their effect on gene expression

The effect of methylation changes on gene expression was evaluated in 17 samples with matching DNA methylation and gene expression data. Although the mechanisms of regulation of gene expression by DNA methylation have not been completely deciphered and positive correlations have also been reported, we focused solely on negative associations (the presence of methylation and loss of expression or *vice versa*).

**Figure 5:**
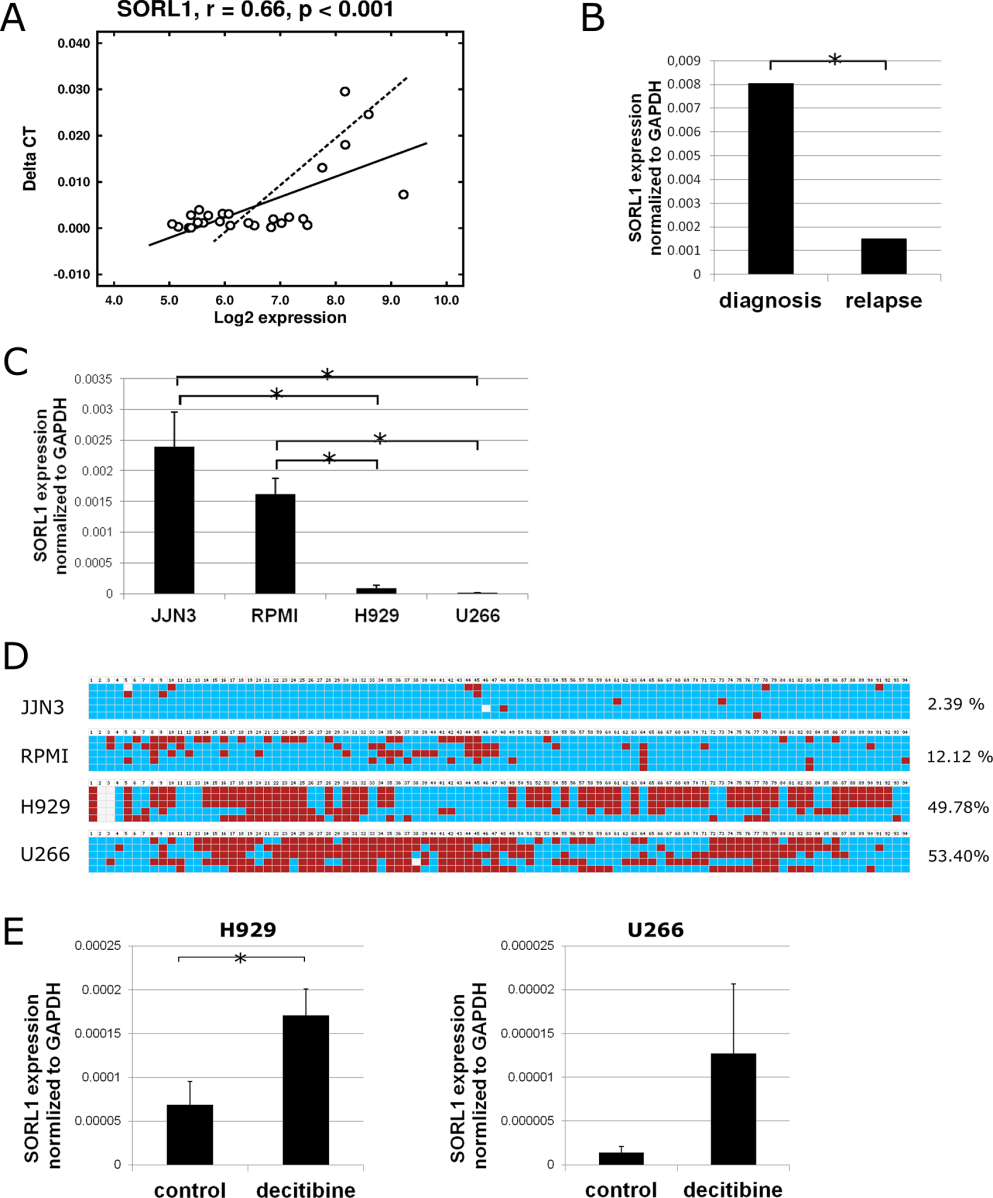
Expression and DNA methylation status of SORL1 gene (**A**) Pearson correlation of *SORL1* gene expression measured by qRT-PCR (Taqman) and by microarrays in 34 MM samples (17 paired samples). (**B**) Expression of *SORL1* gene measured by qRT-PCR at diagnosis and relapse (17 paired samples). (**C**) Expression of SORL1 gene assessed by qRT-PCR in 4 MM cell lines. Results are normalized to the expression of *GAPDH*. Results are shown as ΔC*t* and are the average of three independent experiments. (**D**) DNA methylation status of part of CpG island present in the 5′ UTR region of the *SORL1* gene in H929 and U266 cell lines. Blue square: unmethylated CpG; red square: methylated CpG. Only CpGs are shown. Each line shows one sequenced clone. The percentages indicate percentage of methylated CpGs (average of 5 replicates). (**E**) *SORL1* expression after decitabine (1 μM, 72 h) treatment of H929 and U266 cell lines. Control-cells treated with DMSO. Results are the average of three independent experiments. *p < 0.05 (Student's *t* test).

As a first approach, the list of DMRs was combined with the list of genes whose expression at relapse changed by at least 1.5-fold. An inverse association was only found for *GLT1D1* and *SORL1* genes, so that lower levels of expression of both genes were associated with increased methylation at relapse. Underexpression of *SORL1* at relapse was confirmed by qRT-PCR (Figure 5A and 5B). Next, the relationship between *SORL1* methylation and expression was investigated *in vitro*. To this end, the expression and methylation status of *SORL1* was assessed in four MM cell lines (JJN3, RPMI, H929 and U266). The results showed that the two cell lines (H929 and U266) with the lowest *SORL1* expression levels exhibited methylation in most of the CpGs present in the regulatory 5′UTR region and first exon (Figure 5C and 5D). On the other hand, the two cell lines (JJN3 and RPMI) with high *SORL1* expression levels exhibited lack of methylation in most of the CpGs (Figure 5C and 5D). To test whether DNA methylation repressed *SORL1* expression, the DNA demethylating agent decitabine was used. After decitabine treatment, *SORL1* expression was increased in both MM cell lines (Figure 5E). The results confirmed that *SORL1* expression could be regulated by DNA methylation. Similar results were obtained for *GLT1D1*, although decitabine treatment did not induce a significant increase in *GLT1D1* expression (Supplementary Figure S2).

Subsequently, associations were sought between the expression levels of those genes whose expression changed by at least 2-fold between diagnosis and relapse samples in more than three pairs and the DNA methylation changes in the promoter region and the core promoter of this set of genes. No significant inverse correlations were found using the Pearson algorithm or a “random-effects model fitting” (SIM package).

The lack of significant correlation between DNA methylation and gene expression could be because this relationship exists only in a small set of samples and so its presence may be masked by weak or absent correlations in the majority of samples. This possibility was examined, but we also failed to find other associations between expression and methylation modifications observed at relapse.

### Gene expression profiles associated with relapse

**Figure 6:**
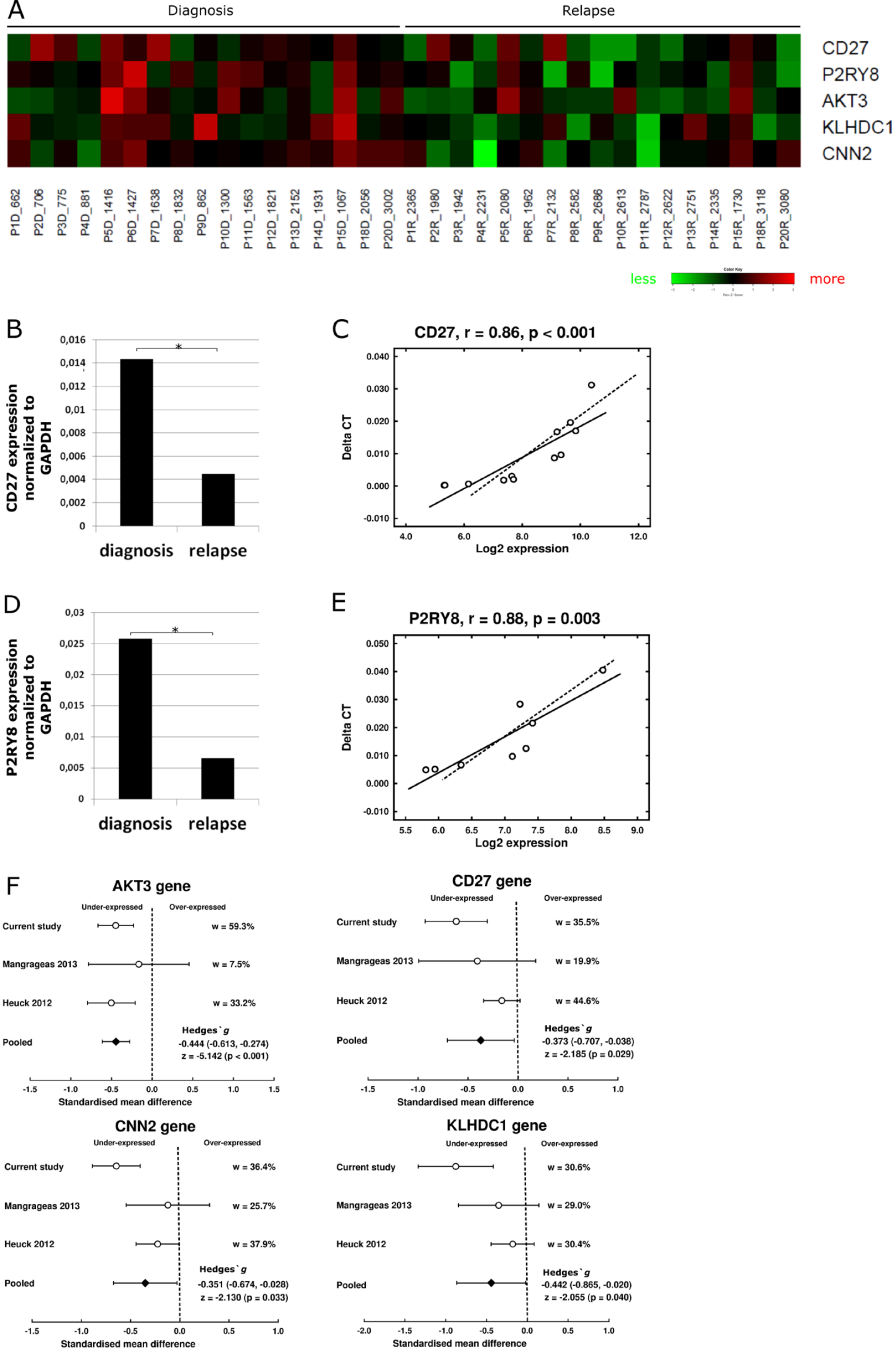
Gene expression profile associated with relapse (**A**) Heat map of the five genes significantly underexpressed at relapse (*q*-value < 0.05). Green- decreased expression, red- increased expression. (**B**) Expression of gene *CD27* measured by qRT-PCR of diagnosis and relapse samples (*n* = 8). (**C**) Pearson correlation of *CD27* gene expression quantified by qRT-PCR (Taqman) and by microarrays (*n* = 8). (**D**) Expression of *P2RY8* gene measured by qRT-PCR at diagnosis and relapse samples (*n* = 12). (**E**) Pearson correlation of *P2RY8* gene expression quantified by qRT-PCR and by microarrays (*n* = 12). (**F**) Meta-analysis of gene expression data. Comparison of standardized mean difference (Hedges' g). w means percentage of weight of each study in the meta-analysis. Effect sizes for individual studies and the combined average are shown in forest plots with their 95% confidence interval (95% CI). For the pooled effect size, the *z* test value and its *p* value were also provided.

Finally, to investigate the transcriptome signature of myeloma cells at relapse, gene expression of paired samples at diagnosis and relapse from 17 MM patients was compared using SAM software. Five genes, *CNN2, P2RY8, CD27, KLHDC1* and *AKT3*, were found to be underexpressed at relapse (*q* < 0.05) as shown in Figure 6A. *CD27* and *P2RY8* expression was further validated by qRT-PCR in sets of six and four paired samples, respectively (Figure 6B–6E). Additionally, we performed a meta-analysis combining our data with two external series of microarray data in which diagnosis and relapse samples were included. The pooled effect sizes of the four genes, *CNN2, CD27, KLHDC1* and *AKT3* (*P2RY8* was not included because of probe set disparity between arrays) showed a negative trend (*g* < 0) indicating a significant underexpression in the relapse samples (Figure 6F).

**Figure 7:**
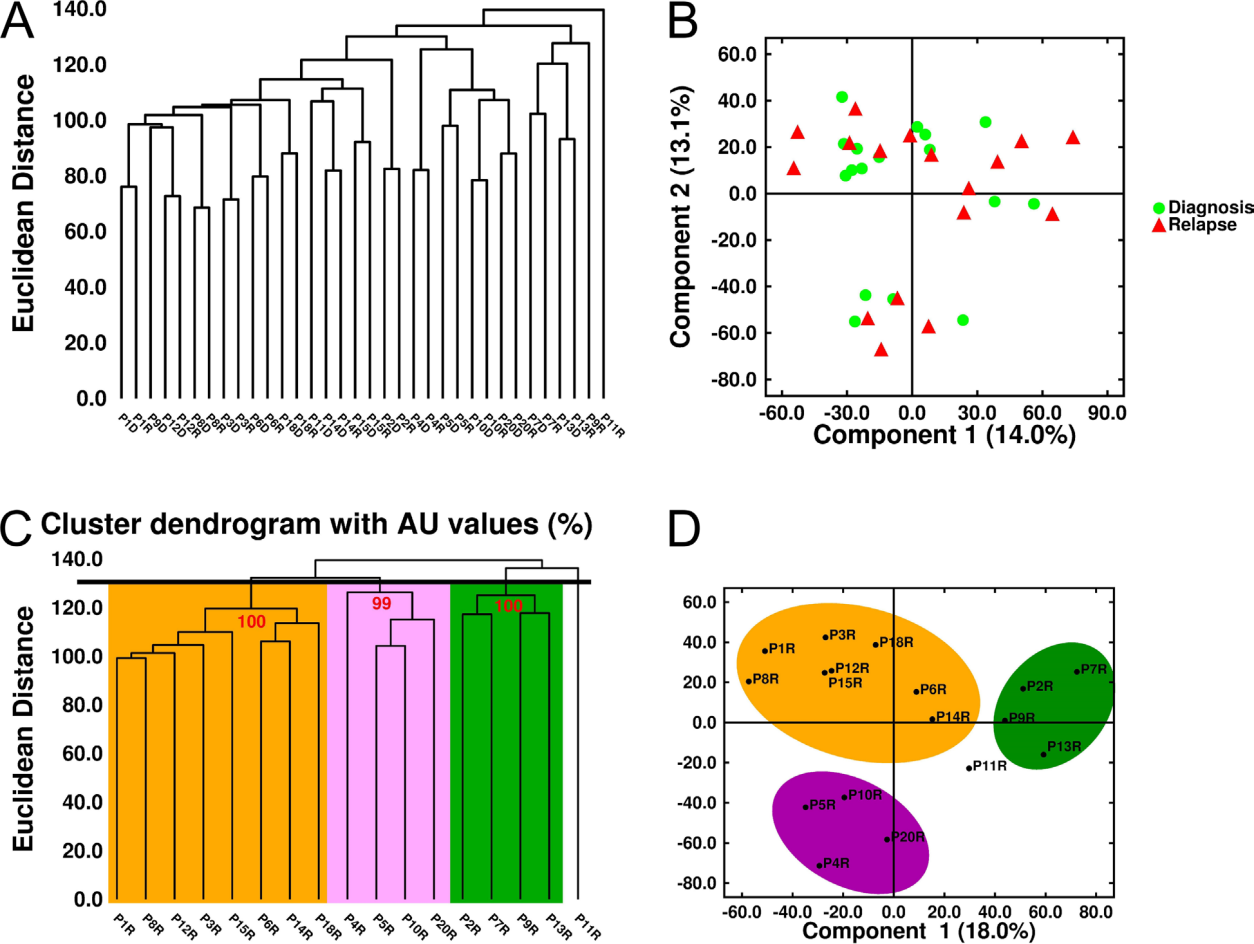
Unsupervised analysis of gene expression in MM samples (**A**) Hierarchical dendrogram and (**B**) Multidimensional scaling of 34 MM samples (17 paired samples) based on the expression of 33297 genes. (**C**) Hierarchical dendrogram plot adjusted to show the AU (Approximately Unbiased) *p*-values (red digits). (**D**) Multidimensional scaling of the 17 relapse samples. Three clusters were identified with a Euclidean distance of ~130.

The low number of differentially expressed genes (DEGs) between relapse and diagnosis led us to investigate the natural structure of gene expression data using an unsupervised approach. Both the multidimensional scaling algorithm and the hierarchical dendrogram showed that samples were mainly grouped as diagnosis-relapse pairs (Figure 7A and 7B). When relapse samples were submitted to hierarchical cluster analysis based on a Euclidean distance of ~130 (Figure 7C and 7D) three groups of eight, four and four samples each were distinguished. SAM analysis was used to identify the DEGs between the relapse samples in each group and the matched diagnosis samples. However, similar to the first analysis, only two genes, *ARHGAP31* and *KIT*, were significantly underexpressed at relapse in the first group; five genes, *PLBD1, ITGAM, IL18RAP, MGAM* and *GPR97* were found to be underexpressed at relapse in the second group, and only the *SNORA5* gene was significantly overexpressed at relapse in the third group.

**Figure 8:**
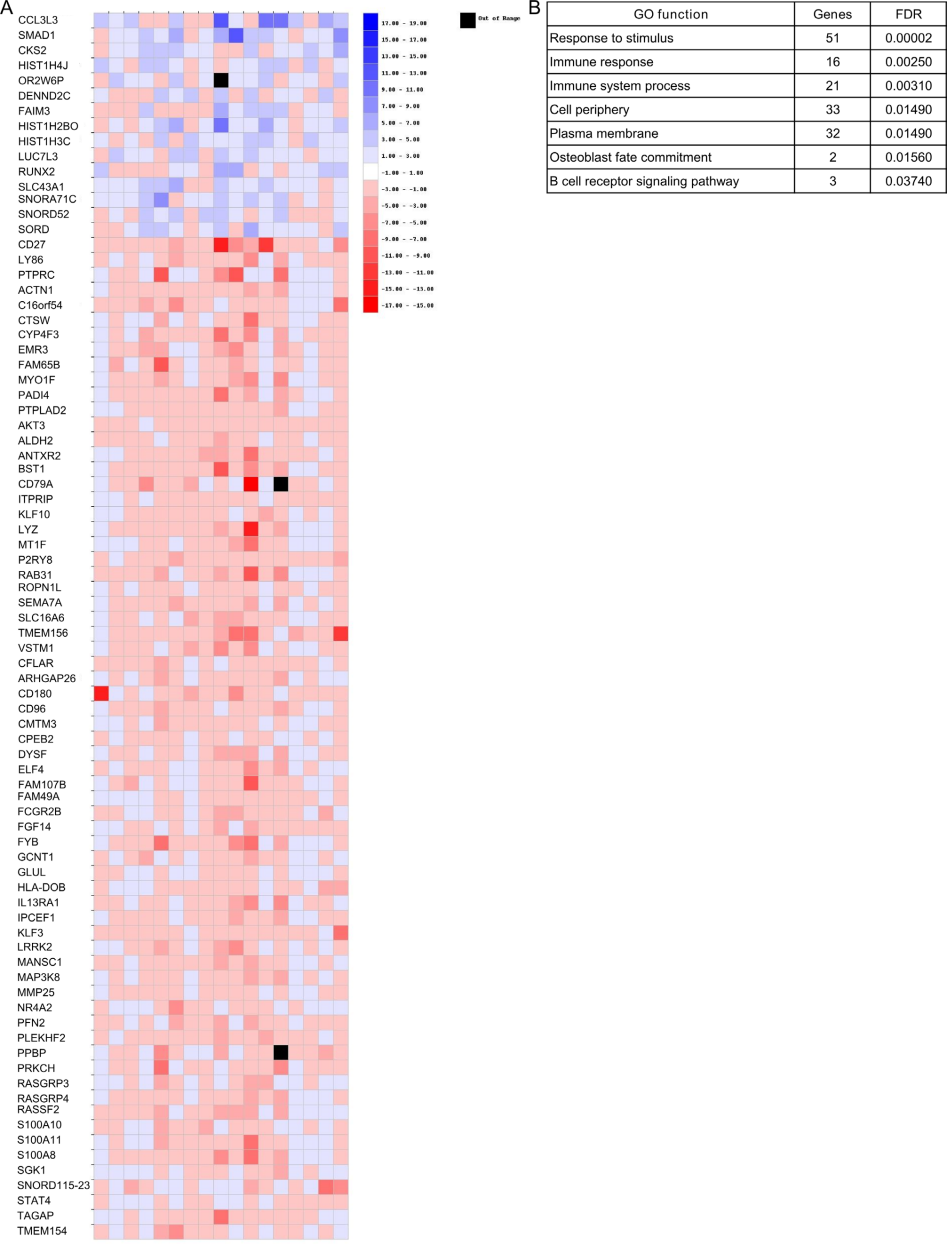
Gene expression changes between diagnosis and relapse in each pair of samples (**A**) Heatmap of those genes that presented changes (|FC| ≥ 2) in the same direction in at least five of the 17 relapse samples. (**B**) Biological processes of genes shown in panel A.

Finally, gene expression changes between diagnosis and relapse in each pair of samples were examined to identify those with a ≥ 2-|fold change| between the two conditions. Only those genes deregulated in the same direction (upregulated or downregulated) in at least five samples were considered. This approach identified 15 overexpressed and 67 underexpressed genes across all samples (Figure 8A). The gene ontology analysis using WebGestalt (Figure 8B) revealed that 15 overexpressed and 67 underexpressed genes were involved in biological processes such as immune system processes (*n* = 21, adjusted *p* = 0.003) and the B cell receptor signaling pathway (*n* = 3, adjusted *p* = 0.04).

## DISCUSSION

Advances in cancer genomic technologies have enabled the genomic landscape of MM to be described in detail. However, the genomic basis of the continuous relapses of myeloma over time, from presentation to end-stage disease remains to be elucidated. Genomic analysis based on copy number abnormalities (CNAs) and massive deep genome sequencing studies has demonstrated that subclonal diversity observed in myeloma cells at the relapse stage often differs from that observed at diagnosis, suggesting that early genetic subclones resistant to initial treatments emerge at relapse. However, it is unknown whether the dramatic differences in the CNA status observed between relapse and diagnosis result in significant changes in the genomic expression profile of myeloma cells. On the other hand, changes in the DNA methylation patterns leading to gene expression deregulation might also contribute to the triggering of MM relapse. The present study aimed to investigate more deeply the genomic changes generated in the transition of myeloma cells from diagnosis to relapse, analyzing not only DNA imbalances but also epigenetic modifications (methylation profiles), and evaluating the influence of both types of DNA modification on the expression phenotype of myeloma cells at relapse.

SNP array analysis revealed different subclonal composition at relapse compared to that at diagnosis in most paired MM samples, consistent with previous reports showing the clonal tiding in MM. Interestingly, the acquisition of chromosomal abnormalities at relapse was significantly more frequent than the loss of lesions present at diagnosis. The CNA most frequently associated with relapse was gains on 8q. The correlation between CNA and the expression level of the genes located on the corresponding DNA regions showed that most of the genes with increased or decreased expression at relapse were not located within the gained or lost DNA regions. Only *PRAME* and *BOP1* overexpression was significantly correlated with gain of the DNA containing these genes. *PRAME* has been found to be expressed in 23% of advanced-stage MM patients [11]. Further studies are needed to ascertain whether *PRAME* expression is associated with a gain of chromosome 22. Gains of 8q24 were only correlated with overexpression of one of the genes, *BOP1*, located at this region. *BOP1* is known to play an oncogenic role in hepatocellular carcinoma by promoting epithelial-to-mesenchymal transition [12]. Although deregulation of *MYC*, a well-known oncogene located in proximity to *BOP1*, has been considered as a late progression event in MM, an association between *MYC* overexpression and 8q24 gains at relapse was not found in this set of samples.

The diversity of changes at relapse might prevent clusters being distinguished with common DNA imbalances when it comes to searching for an associated specific expression signature. Unstable genomes at relapse, with a branching or linear evolution, are more closely associated with high-risk patients than are stable genomes [7]. This prompted us to investigate whether the magnitude of genomic changes, in other words the length of DNA involved in chromosomal imbalances, could influence gene expression patterns. Thus, the 17 paired samples could be divided into two groups on the basis of the length of DNA affected by CNAs at relapse. Interestingly, only modifications in the transcriptome were observed in the group of relapse samples with more than 100 Mb of DNA involved in CNAs. Expression of more than 250 genes was found to be deregulated in this group, with a predominance of underexpressed genes. The IL-8 signaling pathway was the most significantly altered, particularly because of the decreased expression of two IL-8 receptors, *CXCR1* and *CXCR2*. IL-8 plays a critical role in tumor growth, and promotion of cancer invasion and metastasis. In this context, decreased expression of *CXCR1* and *CXCR2* could act as a counterbalancing mechanism leading to the reduction of IL-8 signaling in relapsed MM. The exact role of IL-8 in MM relapse is worthy of further investigation.

Epigenetic modifications form a complex network of inter-dependent mechanisms that are known to be critical factors in cancer development and progression [13, 14]. One of the most widely investigated epigenetic modifications is DNA methylation. In plasma cell dyscrasias, global hypomethylation and gene-specific DNA hypermethylation during the transformation from MGUS to myeloma have been described. However, our study identified no significant modifications in global DNA methylation at the relapse stage. A similar observation was made in acute lymphocytic leukemia in which DNA methylation of some crucial genes like *MDR1, p73, p15 (CDKN2B)* and *p16 (CDKN2A)* was stable in a majority of patients with relapsed leukemia [15]. Nevertheless, further studies with more paired diagnosis-relapse samples from MM patients are needed to confirm this negative result.

A further analysis using an algorithm for DMR detection identified roughly 500 DMR-associated genes between relapse and diagnosis samples. Members belonging to clusters of differentiation such as *CD7*, *CD37* and *CD82* have previously been shown to be regulated by DNA methylation [16–18]. However, the intersection of the list of DMRs and DEGs between relapse and diagnosis did not show significant overlap, which is consistent with the weak association between DNA methylation and gene transcription observed in MM by other groups [19, 20]. Only two DMR-associated genes (*SORL1* and *GLT1D1*) with decreased expression and increased DNA methylation were identified. *SORL1* is a member of the low-density lipoprotein receptor family [21] that has a potential biomarker role in patients with non-Hodgkin's lymphoma [34, 35], and *GLT1D1* (glycosyltransferase 1 domain containing 1) has been reported to be a candidate oncogene in human colorectal cancers with microsatellite instability [24]. Our results using bisulfite sequencing and decitabine treatment showed for the first time that *SORL1* can be regulated by DNA methylation.

Although the analysis of DMRs is relatively automatic and time-saving, DMRs are sometimes thousands of nucleotides distant from the TSS gene, which may represent an obstacle for further data validation. Keeping this limitation in mind, we decided to investigate DNA methylation in specific regions such as the enhancer/promoter zone (−2000 bp from TSS) and proximal promoter (250 bp either side of the TSS), since many regulatory elements are present within 2000 bp [25] and the sequence of proximal promoters are within the region +/− 250 bp surrounding the TSS have the strongest effect on gene expression [26, 27]. By applying this method, a total of 4270 genes with significant change in DNA methylation of the promoter/enhancer region were identified. Despite this high number of differentially methylated genes, no inverse correlation with gene expression changes was detected.

Finally, we also searched for gene expression changes between diagnosis and relapse irrespective of DNA status. Surprisingly, only five genes were significantly underexpressed at relapse. Four of these (*CNN2, P2RY8, CD27* and *AKT3*) were significantly decreased in those relapse samples, with CNAs involving more than 100 Mb of DNA. Low *CD27* expression is an unfavorable marker in MM patients, linked to shorter survival of MM patients [28, 29]. Even the analysis of differential gene expression between the three clusters identified at relapse and the corresponding diagnosis samples did not improve the results. Indeed, few genes were associated with relapse condition. Interestingly, underexpression of *KIT (CD117)* was observed in a set of samples at relapse. This finding is consistent with a previous observation indicating that *CD117* expression by clonal plasma cells confers a favorable prognosis in multiple myeloma [30], and with the reported decrease in *CD117* expression from monoclonal gammopathy of undetermined significance to smoldering and symptomatic MM [31]. The limited changes between the transcriptome of myeloma cells at relapse and diagnosis suggest a broad diversity in the pattern of MM relapse. This prompted us to undertake a pair-by-pair analysis to look for genes that changed their expression in the same direction in more than five pairs of samples. This approach revealed 82 deregulated genes, many of which belong to the B cell receptor signaling pathway and immune system process. Thus, *PTPRC (CD45)*, a protein tyrosine phosphatase whose lack of expression has previously been associated with the phenotype of progressive MM, was downregulated in MM relapse samples [32]. Likewise, *PRKCH*, a member of the protein kinase C family, which plays an integral role in B cell survival and antigenic responses, and the B cell receptor component *CD79a* were also underexpressed at relapse.

To sum up, cross-platform integration of three sets of microarray data revealed that genomic heterogeneity of MM already described at diagnosis remains at relapse with chromosomal imbalances that emerge and disappear in a variegated fashion. The lack of common genomic patterns at relapse would prevent the identification of broad gene expression changes in the relapse condition. Although specific DNA gains and losses were not associated with the respective modifications in the expression of genes located at these regions, an interesting finding was the significant impact of CNAs involving total DNA of > 100 Mb on gene expression, indicating that the more chaotic the genome, the more gene expression changes will be detected. On the other hand, this study showed a very limited effect of methylation changes at relapse on the transcriptome of myeloma cells.

## MATERIALS AND METHODS

### Patients and cell lines

Twenty patients with newly diagnosed symptomatic MM were studied. Clinical information is summarized in Supplementary Table S5. Paired bone marrow (BM) samples were obtained at diagnosis and at first relapse from all the patients. A CD138-positive PC isolation using the AutoMACs automated separation system (Miltenyi-Biotec, Auburn, CA, USA) was carried out in all the BM samples (purity > 90%). DNA was extracted from samples frozen in RLT-Plus buffer using commercially available kits (Allprep Kit, Qiagen, Valencia, CA). DNA quality and quantity were determined using a ND-1000 Spectrophotometer (Nano-Drop Technologies, Wilmington, DE, USA). Total RNA was extracted using an RNeasy Mini Kit (Qiagen, Valencia, CA, USA) following the manufacturer's protocol. RNA integrity was assessed using an Agilent 2100 Bioanalyzer (Agilent Tech, Palo Alto, CA, USA). All patients provided written informed consent in accordance with the Helsinki Declaration, and the research ethics committee of the University Hospital of Salamanca approved the study.

The H929, RPMI and U266 human MM cell lines were obtained from the American Type Culture Collection (ATCC), and JJN3 was obtained from Deutsche Sammlung von Mikroorganismen und Zellkulturen GmbH (DSMZ). Cell culture conditions and decitabine treatment were performed as described elsewhere [33].

Methylation analysis, SNP-based mapping array and expression profiling were performed in 20, 19, and 17 pairs, respectively. Integrated analysis of CNA and gene expression was carried out in 16 patients, and integrated analysis of DNA methylation and gene expression was conducted in 17 patients Supplementary Table S6

### SNP-based mapping array

Genome-wide detection of CNA was carried out using the standard Affymetrix CytoScan 750k assay protocol (Affymetrix, Santa Clara, CA, USA). Briefly, genomic DNA was digested with Nsp I restriction enzyme, ligated to adaptors and amplified by PCR. PCR products were purified and fragmented, and then end-labeled with biotin, denatured, and hybridized to the CytoScan 750k Array. The arrays were processed using the Fluidics Station 450, GeneChip Scanner 3000 7G and AGCC (Affymetrix GeneChip Command Console Software).

### DNA methylation array

DNA methylation was assessed using the Human DNA Methylation 3x720K CpG Island Plus RefSeq Promoter Array according to the standard procedures of NimbleGen Systems, with minor differences. Arrays were scanned in a NimbleGen MS 200 Microarray Scanner (Roche, Basel, Switzerland).

### Gene expression array

RNA labeling and microarray hybridization methods have been previously reported [34]. Briefly, 300 ng of total RNA were amplified and labeled using the WT Sense Target labeling and control reagents kit (Affymetrix, Santa Clara, CA, USA), and then hybridized to Human Gene 1.0 ST Array (Affymetrix). Washing and scanning were carried out using the Affymetrix GeneChip System (Gene-Chip Hybridization Oven 640, GeneChip Fluidics Station 450 and GeneChip Scanner 7G).

Complete microarray data are available from the Gene Expression Omnibus (www.ncbi.nlm.nih.gov/geo/, accession number GSE77540).

### Copy number analysis

The genomic imbalance of 19 paired samples was analyzed using the Chromosome Analysis Suite of Affymetrix (ChAS). CNAs were reported when the following three criteria were attained: a minimum of 25 markers per segment, 100 Kb minimum genomic size and less than 50% overlap with known copy number variants (http://dgv.tcag.ca/dgv/app/home). Copy number-based heatmaps were constructed using the Integrative Genomics Viewer (version 4.3) [35]. Operations on genomic intervals were carried out using the Galaxy suite [36–38]. The copynumber package, implemented in R, was used to “winsorize” [39] and segment the CNA data in order to construct the frequency plots. A threshold log_2_ ratio was set to 0.1 and −0.1 for gain and loss, respectively. The data analysis workflow is presented in Supplementary Table S2.

### DNA methylation analysis

Data from 20 paired samples were extracted using the NimbleScan Software (version 2.5). Three workflow processes were defined using the peak scores, the raw intensity values and the log_2_ ratios as input.

With the first approach, a peak score was estimated from log_2_ ratios. These data were quantile-normalized using the Affy R package [40] and the batch effect was corrected using the ComBat package in R [41]. Peak parameters were set to a width of 750 bp and a minimum of two probes per peak. A peak score cutoff of 2 was established to determine the peak methylation. Comparisons between diagnosis and relapse methylation status, as well as per sample or per chromosome methylation relapse/diagnosis ratios using bar-plots were depicted.

In the second approach, the Comprehensive High-throughput Arrays for Relative Methylation (CHARM) R package was used to identify the DMRs from this data after controlling for the batch effect [42]. All DMRs with fewer than four probes were removed; significant DMRs were defined as those with a value of *p* < 0.05.

The third approach involved the use of the normalized and adjusted log_2_ ratios. Methylation values were assigned to genes at enhancer/promoter and proximal promoter levels based on the mean or the maximum log_2_ ratio methylation values of probes present in these regions. Unsupervised analysis was carried out in SIMFIT (http://www.simfit.org.uk/) using the Euclidean distances and the group average linkage method. Fifty percent of the genes with lower profile variance were removed [43, 44] to perform SAM paired statistical analysis [45]. Variables with a value of *q* < 0.05 were considered to have significant changes in methylation. Gene enrichment analyses were performed using the web tool Webgestalt with the Gene Ontology and Pathway Commons data sources. The data analysis workflow is presented in Supplementary Table S2.

### Gene expression data analysis

Data from 17 paired CEL files were normalized with the RMA algorithm using the Affymetrix Expression Console version 1.3.1.187. Unsupervised analysis was carried out with SIMFIT. Probesets with a low expression level across all samples were deleted in order to enhance the performance of the analysis [46] of the analysis. Statistical comparisons were conducted with the SAM add-in in Excel, selecting the two-class paired statistical option. Only non-duplicated and well-annotated genes that attained a value of *q* < 0.05 in the SAM analysis were reported in this study. We also introduced another method based on the magnitude of the change in gene expression, considering only those genes with a ≥ 2-|fold change| (in the same direction) in at least five pairs. Gene enrichment analysis was carried out using the web tool Webgestalt. The data analysis workflow is presented Supplementary Table S2.

Gene expression meta-analysis was conducted combining our data with two series of GEO (https://www.ncbi.nlm.nih.gov/geo/) microarray data (GSE38627 and GSE37414) in which diagnosis and relapse samples were included. The CMA software version 3 was used for statistical calculations (https://www.meta-analysis.com/) and the SIMIT package. Heterogeneity between studies was tested using the Cochran's Chi-square test (*Q*-test) and the I^2^ statistic. Studies were considered heterogeneous if *p* < 0.05 for *Q*-test [47] and I^2^ value > 50% [48]. Effect size between the 2 groups analyzed for each study were computed using the standardized mean difference (Hedges' *g*). Since the 3 studies showed significant heterogeneity for the genes tested, the meta-analysis calculations were based on random-effects models which takes into account both between-study and within-study variances. Effect sizes for individual studies and the combined average are shown in forest plots with their 95% confidence interval (95% CI). For the pooled effect size, the *z* test value and its *p* value are also provided.

### Analysis of associations between array data

Association studies were conducted using the same approaches for methylation and CNA data, both associated with gene expression data, following three steps. Firstly, global Pearson correlations were calculated using the Psych R package [49]. In order to improve the accuracy we used the fold change (FC) in each pair, as an association parameter in the case of the gene expression and methylation samples, and the ratio difference in the case of the CNA samples. Only those genes with gene expression |FC| > 2 or |FC| > 1.5 in at least 3 pairs were selected for the correlation analyses. In order to adjust *p*-value the false discovery rate (FDR) method was used. Secondly, we used a method called Statistical Integration of Microarrays (SIM) [50, 51] implemented in R. The input matrices are described in the above Pearson procedure. We considered the chromosome level as the dependent region to be analyzed and the FDR as a procedure for multiple testing correction. In the third step, we proceeded to correlate genes pair by pair carrying out analysis procedure based on the |fold change| > 2. The data analysis workflow is presented in Supplementary Table S2.

A detailed description of the data analysis is provided in the Supplementary Methods file.

### *In vitro* validation of epigenetic regulation of SORL1 expression

DNA spanning *SORL1* 5′UTR and first exon or part of CpG island covering *GLT1D1* 5′UTR was amplified by PCR with following primers: *SORL1* forward 5′-GTAGGGAGAATAAGGAGGTGTGTT-3′ and reverse 5′-TCCCCAATAATACCTACACCTAAAA-3′ *GLT1D1* forward 5′-GTAGAAGTAGGATGGGAGTAGGATT-3′ and reverse 5′-TACACTCCAACCTAAATAACACAAC-3′. The bisulfite DNA treatment procedure, cloning of PCR product and data visualization were performed as described elsewhere [33]. To test whether DNA demethylation affected expression of *SORL1*, selected cell lines were treated with decitabine as previously described [33].

## SUPPLEMENTARY MATERIALS








