# Supplementary material for: Integrative analysis of DNA copy number, DNA methylation and gene expression in multiple myeloma reveals alterations related to relapse
Source: Oncotarget. 2016 Nov 2;7(49):80664–79. doi: 10.18632/oncotarget.13025 (PMC5348347; doi:10.18632/oncotarget.13025)
Supplement: Supplementary file 1 [file oncotarget-07-80664-s001.pdf]

Integrative analysis of DNA copy number, DNA methylation and gene expression in multiple myeloma reveals alterations related to relapse

Supplementary Materials

MICROARRAY DATA ANALYSIS

Genomic imbalance analysis

Thirty-eight CytoScan 750 k CEL files corresponding to nineteen paired samples were processed and analyzed in the *ChAS* console (version 3.1.0.15). The unprocessed CEL files were compared against a reference provided by the array manufacturer. This reference consists of 380 microarrays, 284 are HapMap samples and 96 are samples from blood of healthy male and female individuals obtained from BioServe Biotechnologies. Data was analyzed in *ChAS* console using the hg19 built of the Human genome and the Single Sample Analysis (SSA) option. The analysis performed by SSA comprises three steps: a first preprocessing step which performs dual quantile normalization and signal summarization; a second SNP specific step to calculate the allele peaks; and a third copy number (CN) specific step resulting in $\log_2$ ratios and segment calculation, where copy number calls were individually assigned to each marker using a Hidden Markov Model (HMM), and once assigned, they were aggregated into segments if adjacent markers have the same CN state. The median absolute pair-wise difference (MAPD), which measures the array variability in $\log_2$ ratios, was used to assess the array quality, considering as good quality samples those with a MAPD < 0.35. The selected segments were filtered by limiting to the regions with at least 25 markers and 100 kb, and an overlap with known normal CN variation less than 50% (http://dgv.tcag.ca/dgv/app/home). The differences in the number of imbalances found at diagnosis and relapse were tested for significance using the Wilcoxon-Mann-Whitney *U*-test in the *SIMFIT* statistical software (http://www.simfit.org.uk/) version 7.1.7. CN based heat maps were constructed using the *Integrative Genomics Viewer (IGV)* version 4.3 [10]. Comparisons between relapse and diagnosis segments were performed using the *Galaxy suite* [11–13] through the “Operate on Genomic Intervals” tool using the subtract option (Galaxy version 1.0.0) and returning the non-overlapping pieces of intervals. The results of this analysis were used to quantify the whole length of DNA affected by CN alterations (CNA) at relapse but not at diagnosis and vice versa in each sample.

Weighted $\log_2$ ratios from *ChAS* were also processed using the *copynumber* R package [14] version 1.12.0. This package performs a preprocessing step of detection and modification of extreme values through a method called Winsorization [15] and a single sample segmentation step using Piecewise Constant Fitting (PCF) algorithms. Gamma value, which is the penalty for each discontinuity in the curve, was set to 40, and the minimum number of probes allowed in each segment was set to 5. CN frequency plots were constructed using this package, setting the $\log_2$ ratio threshold for gains and losses to 0.1 and −0.1, respectively.

DNA methylation analysis

Microarray scanned images were checked for the quality of the grid alignment, all showing an alignment score < 0.2, which indicates that alignment was within 20% of ideal. Data were extracted from images using the *NimbleScan* Software (version 2.5). This software computes the intensity and the $\log_2$ ratio data of the two channels (635/532) for each feature in each experiment. At this point we defined three workflow processes in which peak scores, raw intensity values or the $\log_2$ ratios were used as input.

To carry out the “peak scores” workflow, the first step was to reduce technical variation. For that purpose, methylation $\log_2$ ratio values stored in GFF files were quantile normalized [1] using the *affy* R package [2] (version 1.50) and the batch effect was adjusted with the *ComBat* R package [4]. In *ComBat* package parametric empirical Bayes frameworks is applied for adjusting batch effects in data. Although originally designed for gene expression data, *ComBat* has also shown good results when used with DNA methylation data [5]. Once adjusted, the next step was to define the peak parameters. We decided to fix a window width of 750 bp, set the minimum number of probes per peak to 2, and the maximum space between nearby probes within the peak to 500 bp. Then, we established a per probe score from the $\log_2$ ratios, which reflects the probability of positive enrichment at or near each probe using a one sided Kolmogorov-Smirnoff test. This test determines whether the probes in the window are drawn from a significantly more positive distribution of intensity $\log_2$ ratios than those in the rest

of the array. Finally, the average *p*-value from all probes comprising the window was assigned to each peak. This value expressed in $-\log_{10}$ is what we called the peak score. These scores should not be interpreted as *p*-values because of several confounding issues such as multiple-hypothesis correction and significant correlation between nearby probes, but rather as relative scores. Once the peak score matrices were constructed, we selected a peak score cut-off ≥ 2 to determine if a peak was methylated. This peak score represents a probability of being a true methylated peak of 99%. The selected peaks were used to compare the methylation status between diagnosis and relapse, as well as per sample or per chromosome methylation relapse/diagnosis ratios.

Regarding the "raw intensity values" workflow, batch effects were removed from these data stored in the XYS files from *NimbleScan* console, using the *ComBat* R package. A new XYS file was constructed with the resulting adjusted values for each channel (635 nm and 532 nm) and each sample. Comprehensive high-throughput arrays for relative methylation (*CHARM*) R package [9] (version 2.18.0) was used to obtain the differential methylated regions (DMR) from this data. This package is specifically designed to analyze two color Nimblegen arrays and consist of two preprocessing steps: the background estimation and adjust, where we used a modified version of the RMA convolution model to remove this artifact, and a normalization step. This last procedure consists of a *within-sample normalization* process carried out using the Loess [9] method, and a *between-sample normalization* process, performed through the quantile normalization technique [1]. This *CHARM* analysis was carried out using the paired mode. All DMR with less than 4 probes were removed, the average percentage methylation difference within the DMR was $\geq 5\%$, and the *t*-statistic *p*-value cutoff was set in 0.05 in order to define significant DMRs. Final DMR output was sorted by *t*-statistic area, which is calculated as the number of probes comprising the DMR multiplied by the average *t*-statistic. Gene enrichment analysis was carried out using the web tool *Webgestalt* [8] (updated on 2013), considering as significant those pathways or functions with at least 2 genes and a *p*-value ≤ 0.05 after Benjamini-Hochberg correction using the data sources, Gene Ontology and Pathway Commons.

In the case of "$\log_2$ ratios" workflow, we used directly the quantile normalized and batch effect adjusted $\log_2$ ratios. DNA methylation data were analyzed at 2 levels according to the probe relative position in the genome: 1) gene promoter level, defined as the region between the gene transcription start site (TSS) and −2000 bp from the gene TSS, and 2) gene core-promoter level, which is the region at +/− 250 bp from gene TSS. Methylation values were assigned to genes at these levels based on the average or the maximum $\log_2$ ratio methylation values of probes present in these regions. Unsupervised analyses were carried out in *SIMFIT* at both levels using hierarchical clustering or multidimensional scaling techniques, with Euclidean distance as the distance measure and group average as linkage method. In order to address the statistical analysis with an optimum number of variables, 50% of genes with lower profile variance were removed [6]. Two class paired statistical analysis of data was conducted on *SAM* "add-in" in Excel [7] version 4.01. Variables with a *q*-value ≤ 0.05 were considered to have significant changes in methylation at each level. Gene enrichment analysis was performed using the significant variables from *SAM* in the web tool *Webgestalt*.

All the features considered in the methylation analysis are represented in the Supplementary Figure S1.

Gene expression analysis

Data from seventeen paired samples were extracted from CEL files and RMA normalized using the *Affymetrix Expression Console* version 1.3.1.187, obtaining a $\log_2$ gene expression level matrix for 17 paired samples. The quality controls and the preprocessing evaluations were carried out using the *AffyQC*_Module through its online implementation in http://www.arrayanalysis.org/. Unsupervised analysis was carried out in *SIMFIT* in order to check the data structure and to test if batch effects were present. Neither artifacts nor known batch effects were detected in our samples. Probesets with a low expression level across all samples, which were considered subject to possible background interferences, were deleted in order to increase the analysis performance. Normalization control probesets present in the Human Gene 1.0st were also removed. We conducted four different approaches to evaluate the changes in gene expression level:

- The first aspect analyzed was the differential gene expression considering all samples. To address this issue, two class paired statistical analysis was performed using the *SAM* "add-in" in Excel. Only non-duplicated and well-annotated genes, which reached a *q*-value ≤ 0.05 in SAM analysis were reported in this study.

- Second, we conducted a paired SAM analysis on the groups obtained from the unsupervised analysis. These groups were defined using a Euclidean distance cut-off value of ~130 as shown in the dendrogram depicted in Figure 7C. The 3 groups obtained in this step were strongly supported by data according the Aproximate Unbiased (AU) *p*-value (> 95%) computed by multiscale bootstrap resampling (H. Shimodaira (2002). An approximately unbiased test of phylogenetic tree selection, Systematic Biology, 51, 492-508.) using the *pvclust* package version 2.0.0 in R. A *q*-value ≤ 0.05 were considered to be statistically significant for these comparisons.

- Third, we introduced a fold change (FC) based gene expression analysis, considering only those genes whose |FC| was ≥ 2 at least in 5 pairs which showed the same change direction in all that pairs.

- As a fourth point, we performed a gene expression analysis on the CNA groups defined after subtraction process with *Galaxy* in the "genomic imbalance analysis" section. These groups were established fitting a two-component Gaussian mixture model using the *mixtools* R package version 1.0.4. The samples were then classified in 2 groups according the length of DNA affected by copy number abnormalities by considering the intersection point between the 2 fitted lines. The SAM Excel "add-in" was used to detect differentially expressed genes between relapsed and diagnosis samples in each CNA group.

Gene enrichment analysis was performed using the output genes from each gene expression analysis with the web tool *Webgestalt*.

Association analysis between DNA methylation or copy number patterns and gene expression profiles

Association studies were conducted using the same techniques for methylation and CNA data, both associated with gene expression data. This process was carried out in three steps in both cases.

On the first step, we integrated our data using global Pearson correlations. We selected this method because is a common technique used in several association studies [16–19]. These correlations were calculated using the *psych* R package [20] version 1.6.4. In order to improve the accuracy, we used the FC in each pair as association parameter in the case of gene expression and methylation samples, or the ratio difference in the case of CNA samples. Only those genes with gene expression FC ≥ 2 or FC ≥ 1.5 in at least 3 pairs were selected for the correlation analyses. The *p*-values were adjusted using the false discovery rate (FDR) method in R with the "p.adjust" function implemented in the *stats* package.

On the second step, we used a method called *Statistical Integration of Microarrays (SIM)* implemented in R [21,22] (version 1.42.0). We chose this method due to its high sensitivity in copy number and expression data integration studies [23]. This statistical procedure identifies associations between two array data sources such as methylation, gene expression or SNP call, based on a random effects model for gene-sets. The input matrices are described in the above Pearson procedure. We considered the chromosome level as the dependent region to be analyzed and the FDR as multi-test adjusting correction. We also used the overlap method which interrogates the overlapping variables between both datasets. The individual associations between each probe in both datasets were calculated as z-scores.

On the third step, we proceeded to associate gene expression with methylation or CNA in a pair by pair procedure, considering only those genes whose expression FC $\geq |2|$ and methylation FC $\geq |2|$ or the weighted $\log_2$ ratio change of CNA $\geq |0.5|$. On a second approach, cutoff values were reduced to expression FC $\geq |1.5|$, methylation |FC| was ≥ 1.5 and the weighted $\log_2$ ratio change of CNA $\geq |0.3|$. The aim of this analysis was to show the frequency of the number of samples in which CNA or methylation change was associated to an expression modification. In the case of CNA this association should be a direct association, while in the case of the methylation this association should be inverse.

REFERENCES

1. Bolstad BM, Irizarry RA, Astrand M, Speed TP. A comparison of normalization methods for high density oligonucleotide array data based on variance and bias. Bioinforma Oxf Engl. 2003; 19:185–193.
2. Gautier L, Cope L, Bolstad BM, Irizarry RA. affy--analysis of Affymetrix GeneChip data at the probe level. Bioinforma Oxf Engl. 2004; 20:307–315. doi:10.1093/bioinformatics/btg405
4. Johnson WE, Li C, Rabinovic A. Adjusting batch effects in microarray expression data using empirical Bayes methods. Biostat Oxf Engl. 2007; 8:118–127. doi:10.1093/biostatistics/kxj037
5. Sun Z, Chai HS, Wu Y, White WM, Donkena KV, Klein CJ, et al. Batch effect correction for genome-wide methylation data with Illumina Infinium platform. BMC Med Genomics. 2011;4: 84. doi:10.1186/1755-8794-4-84
6. Bourgon R, Gentleman R, Huber W. Independent filtering increases detection power for high-throughput experiments. Proc Natl Acad Sci USA. 2010; 107:9546–9551. doi:10.1073/pnas.0914005107
7. Tusher VG, Tibshirani R, Chu G. Significance analysis of microarrays applied to the ionizing radiation response. Proc Natl Acad Sci USA. 2001; 98:5116–5121. doi:10.1073/pnas.091062498
8. Zhang B, Kirov S, Snoddy J. WebGestalt: an integrated system for exploring gene sets in various biological contexts. Nucleic Acids Res. 2005;33:W741–748. doi:10.1093/nar/gki475
9. Aryee MJ, Wu Z, Ladd-Acosta C, Herb B, Feinberg AP, Yegnasubramanian S, et al. Accurate genome-scale percentage DNA methylation estimates from microarray data. Biostat Oxf Engl. 2011; 12:197–210. doi:10.1093/biostatistics/kxq055
10. Robinson JT, Thorvaldsdóttir H, Winckler W, Guttman M, Lander ES, Getz G, et al. Integrative genomics viewer. Nat Biotechnol. 2011; 29:24–26. doi:10.1038/nbt.1754
11. Goecks J, Nekrutenko A, Taylor J, Galaxy Team. Galaxy: a comprehensive approach for supporting accessible, reproducible, and transparent computational research in the life sciences. Genome Biol. 2010;11: R86. doi:10.1186/gb-2010-11-8-r86
12. Blankenberg D, Von Kuster G, Coraor N, Ananda G, Lazarus R, Mangan M, et al. Galaxy: a web-based genome analysis tool for experimentalists. Curr Protoc Mol Biol Ed Frederick M Ausubel Al. 2010;Chapter 19: Unit 19.10.1–21. doi:10.1002/0471142727.mb1910s89

13. Giardine B, Riemer C, Hardison RC, Burhans R, Elnitski L, Shah P, et al. Galaxy: a platform for interactive large-scale genome analysis. Genome Res. 2005; 15:1451–1455. doi:10.1101/gr.4086505
14. Nilsen G, Liestøl K, Van Loo P, Moen Vollan HK, Eide MB, Rueda OM, et al. Copynumber: Efficient algorithms for single- and multi-track copy number segmentation. BMC Genomics. 2012;13: 591. doi:10.1186/1471-2164-13-591
15. Dixon WJ. Simplified Estimation from Censored Normal Samples. Ann Math Stat. 1960; 31:385–391. doi:10.1214/aoms/1177705900
16. Jung S, Kim S, Gale M, Cherni I, Fonseca R, Carpten J, et al. DNA methylation in multiple myeloma is weakly associated with gene transcription. PloS One. 2012; 7:e52626. doi:10.1371/journal.pone.0052626
17. Chin K, DeVries S, Fridlyand J, Spellman PT, Roydasgupta R, Kuo WL, et al. Genomic and transcriptional aberrations linked to breast cancer pathophysiologies. Cancer Cell. 2006; 10:529–541. doi:10.1016/j.ccr.2006.10.009
18. Lee H, Kong SW, Park PJ. Integrative analysis reveals the direct and indirect interactions between DNA copy number aberrations and gene expression changes. Bioinforma Oxf Engl. 2008; 24:889–896. doi:10.1093/bioinformatics/btn034
19. Rhee J-K, Kim K, Chae H, Evans J, Yan P, Zhang BT, et al. Integrated analysis of genome-wide DNA methylation and gene expression profiles in molecular subtypes of breast cancer. Nucleic Acids Res. 2013; 41:8464–8474. doi:10.1093/nar/gkt643
20. Revelle W. psych: Procedures for Psychological, Psychometric, and Personality Research [Internet]. 2015. Available: https://cran.r-project.org/web/packages/psych/index.html
21. Menezes RX, Boetzer M, Sieswerda M, van Ommen G-JB, Boer JM. Integrated analysis of DNA copy number and gene expression microarray data using gene sets. BMC Bioinformatics. 2009;10: 203. doi:10.1186/1471-2105-10-203
22. Goeman JJ, van de Geer SA, de Kort F, van Houwelingen HC. A global test for groups of genes: testing association with a clinical outcome. Bioinforma Oxf Engl. 2004; 20:93–99.
23. Louhimo R, Lepikhova T, Monni O, Hautaniemi S. Comparative analysis of algorithms for integration of copy number and expression data. Nat Methods. 2012; 9:351–355. doi:10.1038/nmeth.1893

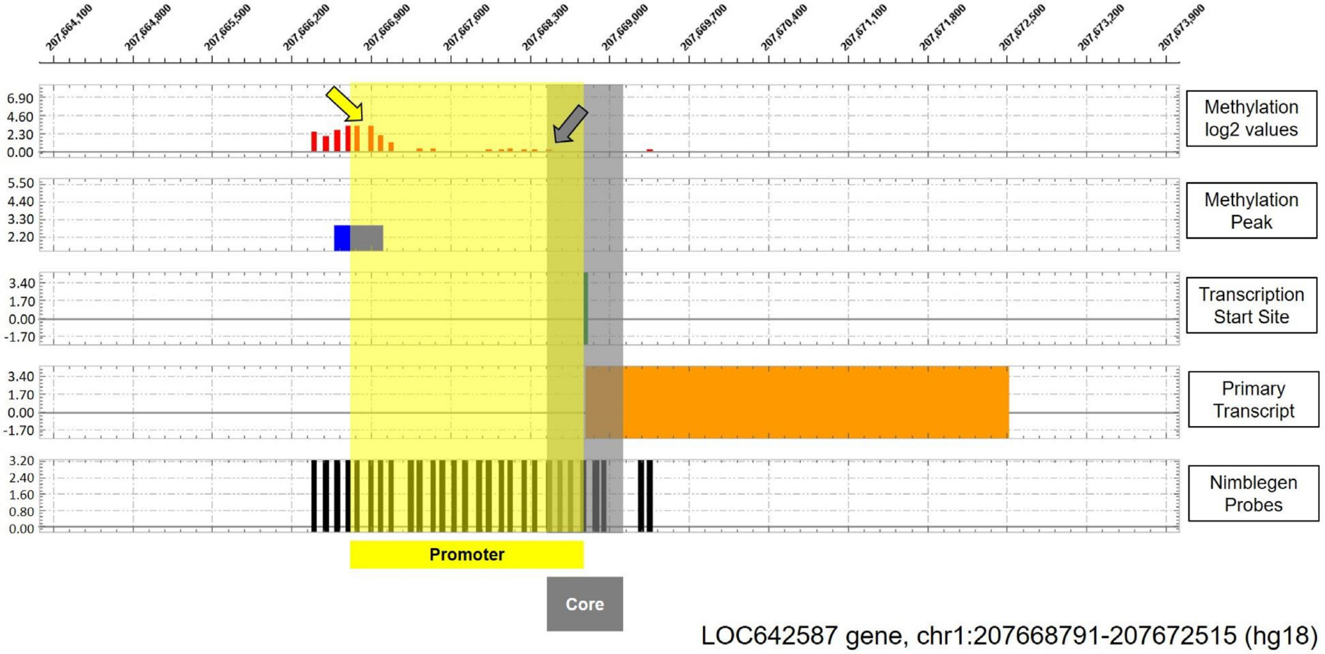

Supplementary Figure S1: Representation of the features present in the methylation studies. The log2 methylation values for each probe in the array are shown as red bars in the top of the figure; the blue, green an orange rectangles represent the methylation peak, the transcription start site (TSS) and the primary transcript for the example gene, respectively. The black bars correspond to the probes of the array. The promoter (−2000 base pairs to TSS) and the core promoter (+/− 250 base pairs from TSS) are depicted as yellow and grey rectangles in the bottom of the figure, respectively. The yellow and grey highlighted areas represent the averaged region when the promoter or the core promoter was analyzed, respectively. The yellow and grey arrows show the maximum of the region when the promoter or the core promoter was analyzed, respectively.

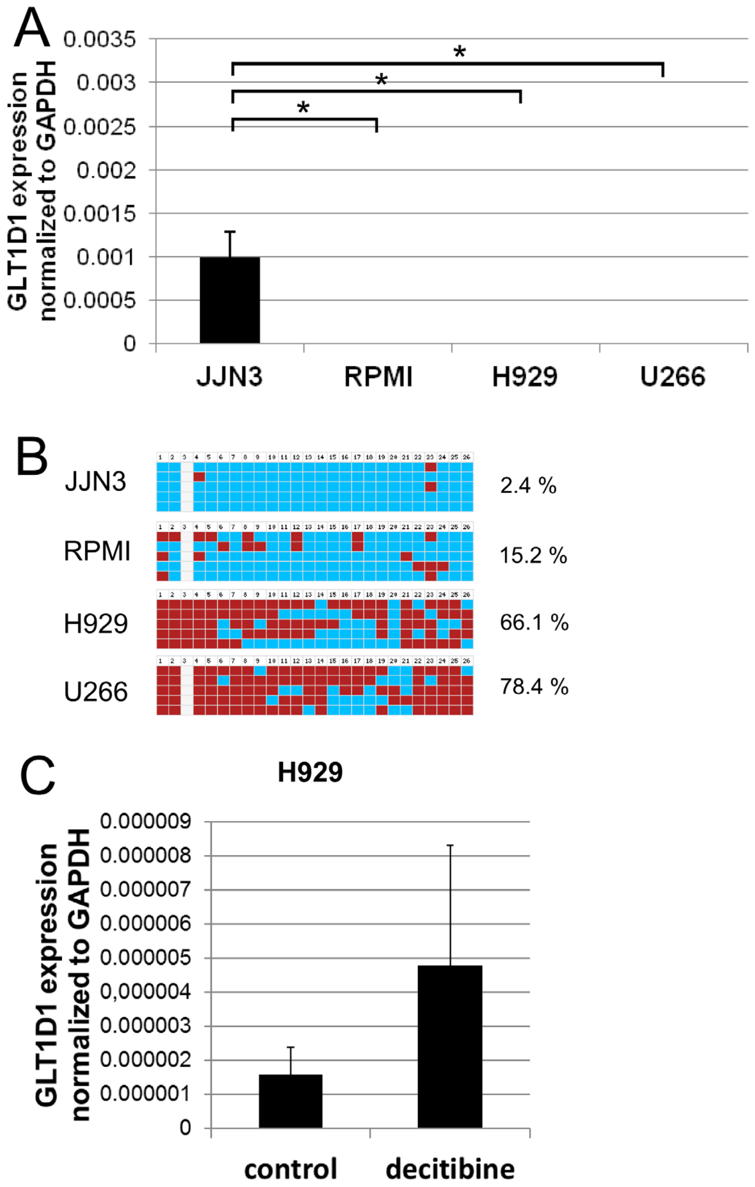

Supplementary Figure S2: Expression and DNA methylation status of GLT1D1 gene. (**A**) Expression of GLT1D1 gene assessed by (Taqman) qRT-PCR in 4 MM cell lines. Results are normalized to the expression of GAPDH. Results are shown as ΔCt and are the average of three independent experiments. *$p < 0.05$ (Student's *t* test) (**B**) DNA methylation status of part of CpG island present in the 5′ UTR region of the GLT1D1 gene in JJN3, RPMI, H929 and U266 cell lines. Blue square: unmethylated CpG; red square: methylated CpG. Only CpGs are shown. Each line shows one sequenced clone. The percentages indicate percentage of methylated CpGs (average of 5 replicates).(**C**) GLT1D1 expression after decitabine (1 µM, 72 h) treatment of H929 cell line. Control-cells treated with DMSO. Results are the average of five independent experiments.

Supplementary Table S1: List of gains and losses observed at diagnosis and relapse in each sample.
See Supplementary_Table_S1

Supplementary Table S2: Summary of bioinformatic strategies used to analyse and interpret microarray data

CNA	Data extraction using ChAS console (Affymetrix)	Segment summary (25 markers, 100 Kb, < 50% overlap with known CNVs)	Relapse *vs.* diagnosis number of segments comparisons: Wilcoxon-Mann-Withney *U*-test			
			Segment heatmap using IGV			
			Relapse vs. diagnosis differences using Galaxy software.			
		Weighted $\log_2$ ratios	Analysis of imbalance frequency using the copynumber package in R			
Methylation	Data extraction using NimbleScan console	"Peak scores" analysis (Methylated region if score ≥ 2)	Batch effect adjust and quantile normalization of $\log_2$ ratio values stored in GFF files. Peak Score calculation.	Methylation absolute differences between relapse and diagnosis		
				Methylation relapse/diagnosis ratio by sample		
				Methylation relapse/diagnosis ratio by chromosome		
		"Raw intensity values" analysis	Batch effect correction of intensity values in XYS files. Loess within-sample normalization and Quantile between sample normalization.	DMR analysis		
		"Log_2 ratio" analysis	Quantile normalization and batch effect removal Variance based filter of probes.	Promoter (–2 Kb from TSS)	Mean of the region	Unsupervised
						SAM paired analysis
					Maximum of the region	Unsupervised
						SAM paired analysis
				Core promoter (+/− 250 pb)	Mean of the region	Unsupervised
						SAM paired analysis
					Maximum of the region	Unsupervised
						SAM paired analysis
Expression	Data extraction using the Expression console of Affymetrix	Gene expression level	RMA normalization	Unsupervised analysis		
				Data background filtering		SAM paired analysis over the whole set of samples (*N* = 34)
						SAM paired analysis in unsupervised analysis based groups
						Gene expression analysis by pair based on fold changes (|FC values| > 2) in at least 5 samples, deregulated in the same direction.
						SAM paired analysis in copy number changes based groups
Association studies	Pearson correlation	Gene selected based on gene expression: FC ≥ 2 or FC ≥ 1.5 in at least 3 pairs	CNA vs. gene expression			
			Methylation vs. gene expression	Promoter (−2 Kb from TSS)		Mean of the region
						Maximum of the region
				Core promoter (+/− 250 pb)		Mean of the region
						Maximum of the region
	Analysis using SIM package	Chromosome level	CNA vs. gene expression			
			Methylation vs. gene expression	Promoter (−2 Kb from TSS)		Mean of the region
						Maximum of the region
				Core promoter (+/− 250 pb)		Mean of the region
						Maximum of the region
	Pair by pair procedure	Approach 1	Gene expression |FC| and DNA methylation |FC|, inverse association			
			Gene expression |FC| CN log2 ratio change |FC|, direct association			
		Approach 2	Gene expression |FC| and DNA methylation |FC|, inverse association			
			Gene expression |FC| and CN log2 ratio |FC|, direct association			

Supplementary Table S3: List of all DMRs detected by Nimblegen methylation array.
See Supplementary_Table_S3

Supplementary Table S4: Summary of genes with differentially methylated regions between relapse and diagnosis samples, detected in a defined DNA range. See Supplementary_Table_S4

Supplementary Table S5: Clinical information of the patients

Patient No/Pair	Age	Gender†	Treatment*	Bortezomib-containing regimen	ASCT**
15122/P1	52	M	VAD	NO	YES
15480/P2	69	M	MPT	NO	NO
16135/P3	60	F	Thal-Dex	NO	YES
16897/P4	58	M	VBAD	NO	YES
20567/P5	69	M	Bd	YES	YES
20653/P6	75	M	VMP	YES	NO
22078/P7	74	F	VMP	YES	NO
22878/P8	76	F	VMP	YES	NO
16780/P9	56	M	VBMCP/VBAD	NO	YES
19986/P10	62	F	VBMCP/VBAD	NO	YES
21552/P11	65	M	VMP	YES	NO
22841/P12	75	F	VMP	YES	NO
25254/P13	60	F	Bd	YES	NO
26794/P14	70	F	VMP	YES	NO
18079/P15	66	M	VBMCP/VBAD	NO	NO
11948/P16	69	M	VTP	YES	NO
26943/P17	60	M	Bd	YES	NO
24605/P18	63	F	VBMCP/VBAD	NO	YES
29159/P19	65	M	Bd	YES	YES
8191/P20	57	M	BenVelPred	YES	NO

†M: male; F: female.
*VAD: Vincristine, Adriamycin, Dexamethasone; MPT: melphalan, prednisone, thalidomide; TD: thalidomide, dexamethasone; VBAD: vincristine, BCNU, doxorubicin, dexamethasone; Bd: bortezomib, dexamethasone; VMP: bortezomib, melphalan, prednisone; VTP: bortezomib, melphalan, prednisone; VBMCP/VBAD: vincristine, carmustine, melphalan, cyclophosphamide, prednisone/vincristine, BCNU, adriamycin, dexamethasone.
**ASCT: stem cell transplantation.

Supplementary Table S6: Summary of performed microarrays

Sample Pair	Methylation array	Expression array	SNP array
P1	YES	YES	YES
P2	YES	YES	YES
P3	YES	YES	YES
P4	YES	YES	YES
P5	YES	YES	YES
P6	YES	YES	YES
P7	YES	YES	YES
P8	YES	YES	YES
P9	YES	YES	YES
P10	YES	YES	YES
P11	YES	YES	YES
P12	YES	YES	YES
P13	YES	YES	YES
P14	YES	YES	YES
P15	YES	YES	YES
P16	YES	NO	YES
P17	YES	NO	YES
P18	YES	YES	NO
P19	YES	NO	YES
P20	YES	YES	YES
